# The diminishing association between adolescent mental disorders and educational performance from 2006–2019

**DOI:** 10.1002/jcv2.12239

**Published:** 2024-05-31

**Authors:** Magnus Nordmo, Thomas H. Kleppestø, Bjørn‐Atle Reme, Hans Fredrik Sunde, Tilmann von Soest, Fartein Ask Torvik

**Affiliations:** ^1^ Department of Educational Science University of South‐Eastern Norway Notodden Norway; ^2^ Centre for Fertility and Health Norwegian Institute of Public Health Oslo Norway; ^3^ Department of Psychology, Norwegian University of Science and Technology Trondheim Norway; ^4^ Department of Psychology PROMENTA Research Center University of Oslo Oslo Norway

**Keywords:** ADHD, adolescent mental health, educational performance, internalizing disorders

## Abstract

**Background:**

A rising prevalence of adolescent mental disorders in the Western world has been widely reported, raising concerns for adolescent development and well‐being. Mental disorders are known to negatively impact educational performance. Yet it remains uncertain whether the relationship between mental disorders and educational outcomes has also changed over time and if the change is more pronounced among high compared to low performing students. The aims of this paper are to (1) describe the change over time in the prevalence of common mental disorders in adolescence; (2) determine whether the change in prevalence of common mental disorders differs between high and low performing students; and (3) assess whether the associations between mental health disorders and educational performance have changed over time.

**Methods:**

To address these issues, this study examines potential shifts in the associations between diagnoses of ADHD and internalizing disorders and educational performance among 843,692 Norwegian students graduating from lower secondary education between 2006 and 2019. We utilize population‐wide register data on ADHD and internalizing disorders from primary and specialist care combined with educational outcomes.

**Results:**

Our analysis revealed a marked rise in ADHD prevalence, from 1.0% in 2006 to 2.6% in 2019. Concurrently, diagnoses of internalizing disorders also increased from 1.9% to 4.2%. This increasing trend in diagnoses spanned across all high school grade point average (GPA) categories, thereby not supporting the notion that the rise is predominantly observed among high‐performing adolescents. Importantly, the strength of the associations of internalizing disorders and ADHD with GPA diminished significantly over time. For instance, the difference between the average GPA standardized score for boys with and without an ADHD diagnosis shrunk from 1.0 in 2006 to 0.73 in 2019.

**Conclusions:**

We discuss various potential explanations for this observation and suggest that changes in diagnostic thresholds is a contributing factor.

## Graphical abstract

In Figure 3, we present the comparative average school performance between individuals diagnosed with ADHD and Internalizing disorders versus those without such diagnoses. Notably, recent birth cohorts demonstrate a convergence in performance, suggesting that the disparity between the two groups has lessened compared to earlier birth cohorts.

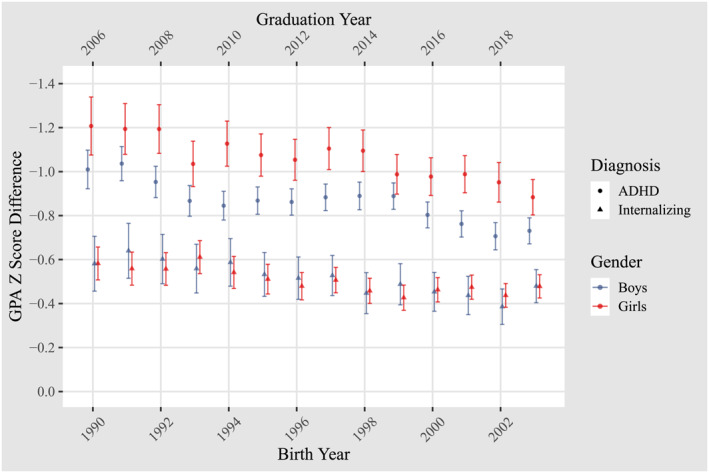

**Key points**

**What’s Known:**

- The prevalence of adolescent mental disorders, including ADHD and internalizing disorders, has been increasing in the Western world.
- Mental disorders negatively impact educational performance, affecting attention, learning, and cognition.

**What’s New:**

- This study demonstrates a significant rise in ADHD and internalizing disorders among Norwegian adolescents.
- The increases in diagnoses are observed across all grade point average performance brackets, with the highest absolute increase among students with the lowest academic performance.
- The strength of the association between these mental disorders and educational performance has diminished over time.

**What’s Relevant:**

- Our results highlight the need for careful diagnostic practices to ensure accurate identification and treatment of mental disorders, avoiding overdiagnosis.
- We encourage further research on the changing dynamics between mental health and life outcomes.

## INTRODUCTION

Adolescence is an important transitional period with identity formation, psychological change, and rapid physical growth. Most mental disorders have their first onset in these years (Solmi et al., 2022) and can have a host of adverse influences on adolescent development and school performance. For example, common mental disorders such as anxiety, depression, and ADHD have detrimental effects on attention, learning, and cognition (James et al., 2021; Loeffler et al., 2019; McTeague et al., 2016) and are associated with poor school performance, school absenteeism, and school dropout (Finning et al., 2019). As a result, mental disorders are negatively associated with educational performance, resulting in a large educational burden of disease (Nordmo et al., 2022). Because educational performance is linked to a wide range of important life outcomes, the potential negative effects of adolescent mental health problems could be far reaching (Davies et al., 2018; Gubbels et al., 2019).

There are many reports of increasing mental disorders and self‐reported mental problems during the last 2 decades from adolescents in the Western world (Collishaw, 2015; Cybulski et al., 2021; Keyes et al., 2019; Mojtabai et al., 2016; Patalay & Gage, 2019), including Norway (Potrebny et al., 2024). Reports show a particularly strong increase in internalizing disorders such as anxiety and depression among girls, whereas boys show a stronger increase in externalizing disorders such as ADHD (Collishaw, 2015; Cybulski et al., 2021). Some have suggested that the driver behind the increasing prevalences of anxiety and depression are high‐performing students, as recent birth cohorts of high‐performing students are reported to be more perfectionistic and under more pressure to excel in school (Flett et al., 2022; Luthar, Kumar, & Zillmer, 2020). According to Luthar, Kumar, and Zillmer (2020), mental health problems in adolescence can be attributed to the pressure of being extraordinarily talented in school. In a Norwegian context, Petersen and Madsen (2023) discuss how ambitious children from an upper‐middle class struggle to reach ambitious goals and suffer as a consequence, while Eriksen (2021) describes how pressure to be the best at school is a source of stress and anxiety. Further, some have argued that this particularly affects girls due to their average higher academic ambitions (Haugan et al., 2021; Högberg & Horn, 2022). Similarly, ADHD is most prevalent in boys and has been shown to be a strong predictor of poor school performance (Sunde et al., 2022) and sensitive to academic pressure (Owens, 2021). To summarize, research has consistently shown increasing rates of self‐reported mental health problems among teenagers in Western countries. Many have suggested that this rise is due to young people, particularly girls, with above average school performance who are pushing themselves harder to achieve more. However, there is a lack of studies that investigate trends in adolescent mental health while also considering school performance.

One perspective on the reports of rising prevalence rates is that they reflect a genuine increase in mental disorders. This viewpoint suggests that recent birth cohorts, born after 1990, are more exposed to risk factors negatively affecting mental health, such as the widespread adoption of social media in a vulnerable age (Potrebny et al., 2024; Twenge, 2020), contributing to an increase of internalizing disorders. There are also speculations that rising rates of ADHD is caused by an increasing exposure to environmental chemicals (Moore et al., 2022). An alternative view on rising rates of mental problems sees the phenomenon as an artifact of changing conceptualizations and perceptions of disease (Brinkmann, 2016; Jackson & Haslam, 2022; Paris, 2020). This shift could manifest in a declining threshold for what is considered pathological. To what extent increasing rates of internalizing disorders and ADHD reflect a genuine increase or a change in threshold is difficult to explore empirically. However, it is valuable to ascertain whether the negative impact of a mental disorder has also changed in parallel with increasing prevalences. We propose educational performance as a particularly important outcome to assess, given the influence of common mental disorders on cognition, sleep, and energy, all of which affect learning and educational performance. Should the association between educational performance and mental disorder weaken over time, this would have significant ramifications for how researchers and policy workers respond to the increasing prevalence of mental disorders. A change in this association could be caused by multiple processes such as increased implementation of effective treatment or positive educational reforms. Another possibility is that there has been a shift in diagnostic thresholds. Unfortunately, studies examining the stability of associations between mental disorders and correlated outcomes across time are sparse. To our knowledge, there are only two studies with cohorts born later than 1990, originating in the Avon Longitudinal Study of Parents and Children (ALSPAC) and the Millennium Cohort Study (MCS). Gage and Patalay (2021) found an increase in the association between self‐reported mental health at age 14 and health‐related behaviors – such as smoking, BMI, substance abuse – when comparing UK cohorts born in 1991/92 and 2000/02. Sellers et al. (2019) compared the same cohorts with respect to parent‐rated adolescent mental health and educational attainment at age 16 and found an increased association in the recent cohorts. Both studies reported increased mental health problems for the recent birth cohorts.

In sum, while many investigations show an increase in adolescent mental disorder and complaints, it is unknown to what extent this trend reflects a genuine increase in pathology or a lowered threshold for what constitutes a diagnosis or complaint. A strategy for investigating this issue is to examine change or stability in how outcomes are associated to mental disorder diagnoses across time. Currently, no temporally informed study relates common mental disorders as measured by primary care diagnoses to educational outcomes. Building on this, we first investigate whether diagnoses of internalizing disorders and ADHD have become more common, as current trends suggest. Second, we will assess whether the associations between diagnoses and school performance have changed over time. This is a crucial step in understanding how mental health diagnoses may affect academic performance and the potential shifting dynamics of this relationship. Third, we will examine time trends in the prevalence of diagnoses in relation to school performance. Specifically, we aim to discern whether high‐performing students in particular have become more prone to develop internalizing disorders, as some suspect. This final point of inquiry will allow us to analyze the intersection of mental health and academic achievement, offering insight into whether the most successful students are paying a high price for their accomplishments.

## METHODS

### Sample

We used administrative register data comprising all individuals born in Norway between 1990 and 2003 and who graduated from primary education the year they turned sixteen (2006–2019; *N* = 843,692). Among adolescents who did not emigrate and who were not diseased before the age of sixteen, we find that nearly all (96%) graduated from primary education the year they turn sixteen. We grouped all individuals in their respective birth cohorts, that is, the year they were born. To ascertain diagnoses of mental disorders we used the national register for reimbursement of primary care physicians (Norwegian Control and Payment of Health Reimbursements Database, KUHR). We selected this timespan to include all years of available diagnostic data. We also utilized data from specialist‐care services from the Norwegian Patient Register (NPR). This included data from both regional outpatient clinics and subsidized contract specialists. We had access to specialists‐care data in the period 2008–2019. The total number of included patients in these analyses was *N* = 752,565. Note that the time frame of our investigation does not overlap with the COVID‐19 pandemic, as the first case was registered in Norway in February 2020. Data on school performance was retrieved from the National Educational Database (NUDB). This work is part of the REMENTA project and was supported by the Research Council of Norway (#300668). This work was partly supported by the Research Council of Norway through its Centres of Excellence funding scheme, project number #262700. Tilmann von Soest's work with this article was supported by a grant from the Research Council of Norway (grant # 300816).

### School performance

Norwegian students are evaluated at the end of 10 years of compulsory education in a range of school subjects. All numeric grades have marks from 1 to 6, where six is best. The GPA is calculated as the average of all final‐year teacher evaluated grades and externally graded exams and is later used for ranking students applying for admission to upper secondary education. It is not possible to fail the compulsory education in Norway and there is no grade retention. All received grades are included in the GPA score, including those that would be considered a failing grade at a higher level of education. Consequently, nearly all students have a valid GPA. We standardized the Grade Point Average (GPA) score (mean = 0, SD = 1) for each graduation yearly cohort. This was done to avoid grade inflation confounding the comparisons across cohorts. Data from Statistics Norway (2023) shows that Norwegian GPA have gradually increased from 3.95 in 2009 to 4.24 in 2023. To explore the change in prevalence of mental disorders with respect to GPA, we divided the GPA into quintiles.

### Diagnoses of mental disorders

Primary care diagnostic information is coded according to the International Classification of Primary Care, 2nd edition, (ICPC‐2: WONCA, 2005), by general practitioners treating the patients. Norwegian primary care services are free of charge for children and adolescents under sixteen. Due to economic incentives, it is unlikely that visits to general practitioners and subsequent diagnosis are not reported. Our goal was to include relatively common adolescent mental disorders that are known to have a negative association with educational performance which left us with internalizing disorders and ADHD. We combined three diagnoses to encompass internalizing disorders: P74 Anxiety Disorder, P76 Depression, and P79 Phobic Disorder. We used P81 Hyperkinetic Disorder to define ADHD. Specialist care diagnostic information was coded using the same scheme but with diagnoses from the International statistical Classification of Diseases and related health problems 10 (ICD 10: World Health Organization, 2004). We defined internalizing diagnoses as F321, F331, F320, F330, F341, F401, F412, F410, F411, F419, and F431 and ADHD as F90.0 and F90.1. We use the colloquial term common mental disorders to describe internalizing disorders and ADHD. We dummy‐coded diagnosis as present (1) if there was a registered diagnosis the year of graduation, or not present (0) if no diagnosis was registered the year of graduation. There are substantial overlaps between diagnoses from primary and specialist care as diagnoses are carried over from specialist care into primary care. In the case of ADHD, primary care general practitioners refer to specialist care for a diagnostic assessment. If the patient qualifies for the diagnosis, then he or she is referred back to the general practitioner for treatment and follow‐up. This procedure entails that most specialist care diagnoses carry over to primary care but not vice versa.

### Statistical analyses

To capture the relationship between mental disorders and educational performance over time, we regressed GPA scores on each mental disorder, the yearly cohort, gender, and interaction terms between mental disorder and cohort. Specifically, we estimated the following multivariate regression model:

$$GPA_{i}=\beta_{0}+\beta_{1}\left(D_{i}\right)+\beta_{2}\left(C_{i}\right)+\beta_{3}\left(G_{i}\right)+\beta_{4}\left(D_{i}\times C_{i}\right)+\varepsilon_{i}$$

D:DummycodedDisorderStatus(Internalizing&ADHD),C:Cohort,G:Gender(0Male,1Female)

We coded cohort (birth year) as zero for the adolescents born in 1990, 1 for 1991, 2 for 1992 et cetera. Similarly, we started the specialist care analyses by coding cohort 1992 as zero. Both disorder status and gender were dummy coded into dichotomous variables. The outcome from this model represents GPA conditioned on disorder, cohort, gender and the interaction for disorder × cohort, as shown in Figure 3. A significant interaction term between cohort and disorder indicates a significant change in the association between mental disorders and school performance across time. To assess the potential for a non‐linear change over time, we estimated and plotted bivariate associations between GPA and mental disorders separately for each year. All confidence intervals are specified at the 95% level.

We did not consider comorbidity between internalizing and ADHD in our analyses. Given the relatively low prevalences and gendered structure, where boys are more likely to receive ADHD and girls internalizing disorders, there was limited comorbidity. Of the boys with either an internalizing or ADHD diagnosis, or both (*n* = 16,992), only 414 (0.02%) received both. Similarly, girls who received either diagnosis or both (*n* = 17,587), only 454 (0.03%) received both.

We calculated the prevalence of each disorder in each graduation year for boys and girls separately. To assess change in prevalence of disorders according to academic achievement, we categorized students into GPA quintiles, ranging from the first quintile (lowest grades) to the fifth quintile (highest grades), and stratified by gender. Subsequently, we merged the second, third, and fourth quintiles, resulting in the formation of three distinct groups: the bottom 20%, the middle 60%, and the top 20% of students.

## RESULTS

### Prevalence changes

**FIGURE 1:**
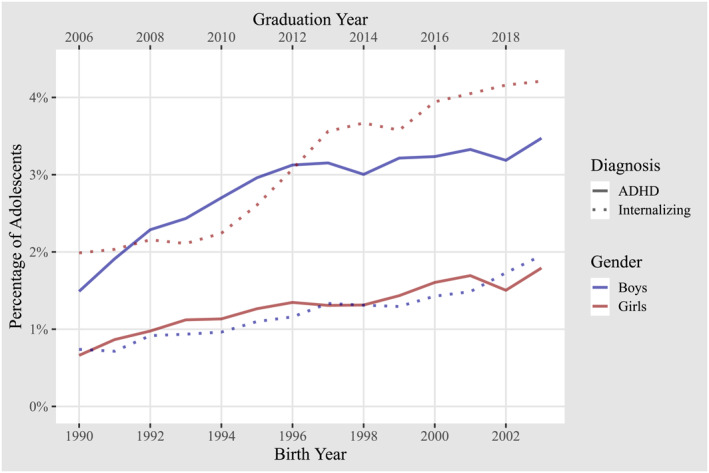
Annual primary care diagnosis prevalence at age 16. The figure shows the proportion of adolescents receiving a primary care diagnosis at age 16, as a function of gender and cohort. Two different line types distinguish between internalizing disorders (dotted) and ADHD (solid). The color of the line represents the gender. The bottom *X* axis label shows the birth year and the top *X* axis label shows the corresponding graduation year of the cohort.

Our analysis revealed a substantial increase of diagnoses of internalizing disorders and ADHD in primary care, with prevalence rates more than doubling between 2006 and 2019, as depicted in Figure 1. For girls the rate of internalizing diagnoses increased from 1.9% to 4.2%, whereas rates of ADHD diagnoses increased from 0.7% to 1.8%. For boys, the rate of internalizing diagnoses increased from 0.7% to 1.9%, whereas rates of ADHD diagnoses increased from 1.5% to 3.5%. Supplemental Figure S1 and Supplemental Table S1 demonstrate that using the data obtained from specialized care give similar results, albeit with a lower total number of cases.

### Mental disorder and school performance

**FIGURE 2:**
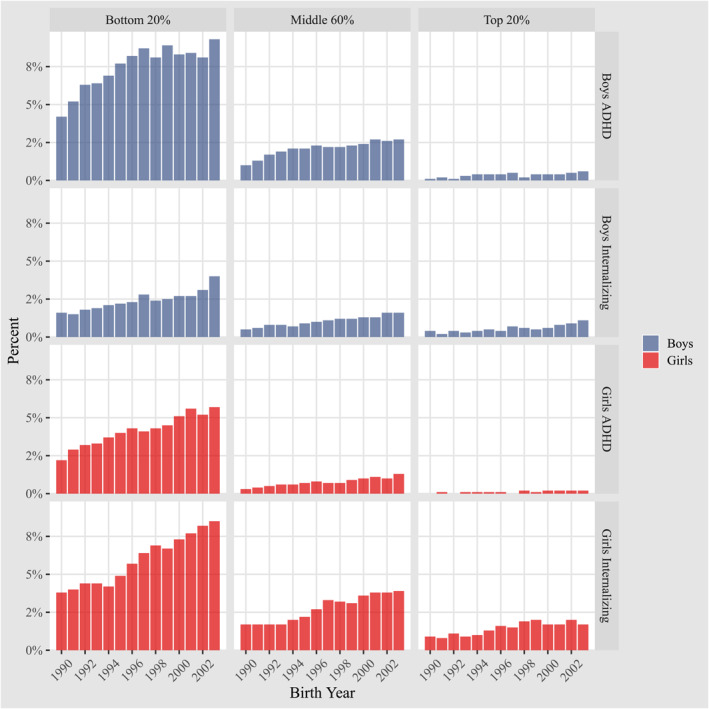
Prevalence by gender, disorder, and GPA group. The figure shows the proportion of adolescents receiving a primary care diagnosis at age 16, as a function of cohort, GPA performance, diagnosis and gender. GPA performance is divided into three groups representing low, average, and high school performance.

As illustrated in Figure 2, the increase in cases of internalizing disorders and ADHD roughly corresponds to the total number of cases seen in each GPA category. This means that categories with fewer initial cases experience a smaller absolute increase but a similar relative proportion increase, compared to categories with a larger initial number of cases, which show a larger absolute but comparable relative increase. However, several patterns are worth noticing. First, the absolute prevalence and prevalence increases were higher within the lowest quintile of school performance. For example, the lowest GPA quintile showed an increasing rate of internalizing diagnoses among girls from 3.8% to 8.5%, while rates in the highest quintile increased from 0.9% to 1.7%. Second, the relative increases were similar across the different GPA groups. For example, girls internalizing in the bottom quintile more than doubled, while girls in the top quintile nearly doubled from approximately 1% to 2%. Third, while the strongest increase in ADHD prevalence was observed for birth cohorts between 1990 and 1995, internalizing disorders increased particularly strongly in birth cohorts from 1995 and onwards.

### Changing associations over time

The results from our regression model indicate that the GPA difference between those with and without ADHD decreased during the observation period with 0.013 *z*‐scores for each year (*p* < 0.001). Over the 13‐year study period, this adds up to a decrease in the difference between those with and without an ADHD diagnosis of 0.17 *z*‐scores.^1^ To put this figure into context – across both girls and boys – the model implied negative effect of ADHD on GPA in the 2003 cohort was 16% less severe compared with the 1990 cohort.^2^ We further found that the difference between those with and without an internalizing disorder shrank with 0.012 *z*‐scores for each year (*p* < 0.001). This adds up to a difference of 0.16 *z*‐scores in the 13‐year study period. This entails a model implied reduction of 27% in the difference between those with and without an internalizing diagnosis from 1990 to 2003.^3^ The main results are shown in Table 1.

**TABLE 1:** Primary care regression models.

	ADHD			Internalizing	
	Coefficient	Standard error		Coefficient	Standard error
Intercept	−0.699 ***	(0.004)	Intercept	−0.742 ***	(0.004)
ADHD	−1.043 ***	(0.015)	Internalizing	−0.597 ***	(0.015)
Cohort	0.006 ***	(0.000)	Cohort	0.005 ***	(0.000)
Gender	0.483 ***	(0.002)	Gender	0.507 ***	(0.002)
ADHD × Cohort	0.013 ***	(0.002)	Internalizing × Cohort	0.012 ***	(0.002)
*N*	843692		*N*	843692	
*R* ^2^	0.084		*R* ^2^	0.071	

***p < 0.001.

**FIGURE 3:**
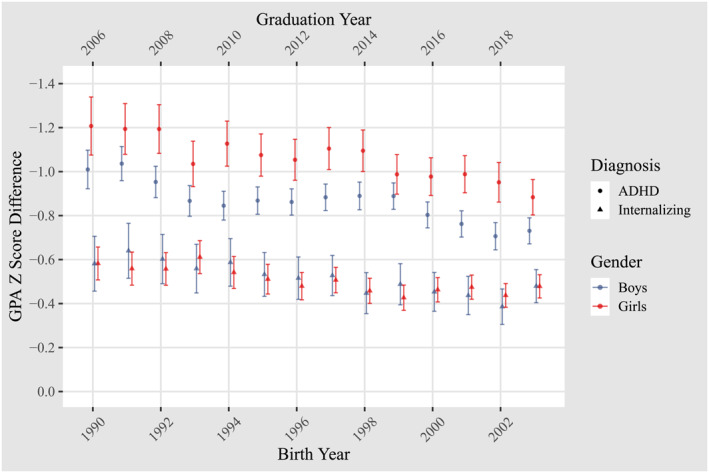
Bivariate associations between mental disorder and GPA across yearly cohorts.

See Figure 3 for plot of all bivariate associations. Similar effects were found when utilizing specialist care diagnoses as shown in Supplemental Figure S1. See Supplemental Figure S2 in the appendix for descriptive plots of average GPA values in both standardized and unstandardized form.

## DISCUSSION

Our study is consistent with an expanding body of research that demonstrates a rise in adolescent diagnoses of internalizing disorders and ADHD. In absolute terms, the increasing prevalence in internalizing and ADHD diagnoses was particularly pronounced among the low‐achieving students, although in relative terms, the increase within each achievement group was similar. Concurrently, we observed an overall decrease in the association between these disorders and educational performance. This was the case for both primary and specialized care diagnoses.

### Adolescent mental health across time

While many studies show alarming increases in mental health problems in adolescents, there is undoubtably heterogeneity across studies. The referenced research on adolescent mental health is categorized into three types: the majority using surveys by parents or adolescents (Collishaw, 2015; Keyes et al., 2019; Mojtabai et al., 2016; Patalay & Gage, 2019; Potrebny et al., 2024), a second group, including our study, employing diagnostic register data (Cybulski et al., 2021), and a third utilizing clinical diagnostic interviews (Polanczyk et al., 2014; Sawyer et al., 2018). Studies in the first two groups consistently report an increase in mental health problems or disorders, but those in the third group do not. This contrasting evidence is compatible with the hypothesis that adolescents are more attentive to their mental health and that increasing availability of treatment might lead to an apparent increase in mental health problems. This change in self‐evaluation might not be present when assessed in standardized clinical interviews. It should also be noted that it is reasonable to assume different prevalence changes in studies originating in different countries using different time spans.

More access to treatment has been shown to increase rates of diagnoses and lead to subjectively worse health, known as the paradox of health (Barsky, 1988; Madsen, 2021; Ormel & Emmelkamp, 2023; Ormel et al., 2022). In recent times, Norway has placed great emphasis on upscaling Norway's treatment capacity for adolescent mental disorders (Research Council of Norway, 2009). According to Statistics Norway, there has been a gradual increase in clinical psychologists employed in state owned hospitals from 2008 (0.7 per 1000 citizens) to 2019 (1.13 per 1000 citizens). Similarly, the Increased Access to Psychological Healthcare Initiative has improved treatment availability (Lervik et al., 2020) in the same timeframe. Following a recent expansion of the Finnish adolescent psychiatry services, Holttinen et al. (2022) tentatively concluded that the perceived need for treatment was reconceptualized with increased access to treatment so that adolescents classified themselves as mentally ill in situations which in earlier decades were interpreted in some other way. This is also consistent with data from US cohorts where Johnson (2021) assessed a wide range of birth cohorts across the period 1997–2017. While the average self‐reported distress was similar across the birth cohorts, the treatment‐seeking was much greater for recent cohorts. Similarly, a recent study of U.S. adolescents with no treatment history for mental disorders found that the youngest cohorts, those born 2000–2002, reported the highest need for treatment, compared to older cohorts (Askari et al., 2022). According to a recent Swedish qualitative investigation, feeling unwell is the “new normal” (Hermann et al., 2022). These empirical findings are parallel to the theoretical work of Brinkmann (2016) who posits that modern adolescents themselves advocate for the pathologizing of mental distress, as they predominantly interpret suffering through a medicalized lens. This perspective contrasts alternative perspectives, such as those offered by religious doctrines or class‐struggle ideologies, where suffering is regarded as a normative, inevitable part of existence.

### The association between mental disorder and educational performance

Our finding of an approximately linear decrease in the association between mental disorders and educational performance are in contrast with three UK studies (Gage & Patalay, 2021; Sellers et al., 2019; Thompson et al., 2021) who reported increased clustering of mental health symptoms with low educational achievement, adverse health outcomes, and substance abuse in recent cohorts. A plausible reason for this disconnect is that we used primary care diagnosis, while the UK studies utilized self‐ or parent‐reports of mental health symptoms. As reported above, the literature suggests a divergence between ratings of subjective experience and a diagnostic assessment based on a structured interview with a healthcare professional.

The diminishing association between common mental disorders and educational performance in adolescence may be attributed to many distinct but overlapping processes. For example, the threshold for attaining a mental disorder diagnosis in Norwegian primary and specialist care may have decreased during the study period, in parallel with the expansion of adolescent mental health services. This argument is based on the observation that admittance to subsidized treatment is prioritized based on severity. Given this scenario, each successive yearly cohort has a reduced threshold for diagnoses, as more adolescents are treated year by year. Consequently, individuals with less severe symptoms are increasingly likely to be diagnosed with an internalizing disorder or ADHD. This possibility is consistent with the considerable increase in the prevalence of diagnoses and the continuous nature of mental disorders (Conway et al., 2021). Starting from the hypothesis that the threshold for treatment has decreased, the diminishing associations could be attributed to two distinct consequences. Firstly, the increase in diagnoses and treatment coverage could cause educational benefits, both by psychotherapeutic and educational interventions. Specifically, the detrimental impact of ADHD could be lessened by increased use of medication which has seen a gradual increase between 2000 and 2013 (Lillemoen et al., 2012; Raman et al., 2018). However, is seems somewhat unlikely that treatment of internalizing disorders has resulted in large positive educational gains, given the discouraging research literature on the subject (Baker et al., 2021; Cuijpers et al., 2023). Secondly, a lowered diagnosis threshold can cause a decreasing association between disorder and educational performance directly. If the less severe end of the healthy‐to‐disorder distribution is diagnosed – and given a link between the severity of the disease and a detrimental educational effect – then it follows that lowering the threshold would result in a decreasing association between disorder and GPA. Other potential processes may as well explain the reduction in associations. For example, the increasing prevalence could lower stigma around mental disorders which could serve as a relief, lessening the negative impact of mental health problems on educational attainment.

In parallel with increased prevalences of adolescent internalizing, many countries observe an increase in indicators of social inequalities, such as the distribution of wealth and income. This is followed by a rise in social inequality in the Norwegian education system (Sandsør et al., 2023), whereby the difference in performance between children form advantaged and disadvantaged backgrounds have increased over time. Concurrently, we found increased differences in mental health between children with low versus high GPA. This could indicate that society is becoming more heterogenous in terms of not only economy but also mental health.

### School achievement and mental disorders

Contrary to some (Flett et al., 2022; Luthar, Kumar, & Zillmer, 2020; Stentiford et al., 2021), we did not find that the increase in common mental disorders were attributable to high‐achieving students or high‐achieving female students in particular. Instead, we found that the bottom quintile of the GPA distributions had the highest prevalence and largest absolute increase in both internalizing disorders and ADHD. This is consistent with well‐established findings that more low‐performing students have mental disorders (Nordmo et al., 2022). In terms of relative increases, all GPA groups saw similar levels of increasing prevalence rates. For example, while boys' ADHD in the bottom quintile saw a doubling from 4% to 8%, girls ADHD in the top quintile GPA group saw a doubling from 0.1% to 0.2%. The finding that most of the internalizing disorders are found in low‐performing students go against the notion that perfectionism or academic expectations among high performing students are the main driver of increased mental disorders (Eriksen, 2021; Flett et al., 2022; Luthar, Kumar, & Zillmer, 2020). Increasing academic pressure to excel might improve school performance while potentially harming mental health (Högberg et al., 2020). This may be particularly detrimental for students who perform poorly despite high expectations. High‐performing students may experience increased pressure, but this can be offset by the advantages of high performance. Nonetheless, it's important for teachers and healthcare professionals to recognize that mental health issues are more prevalent among students with lower academic performance.

### Strengths and limitations

This study has several strengths. It uses data from the entire population of Norwegian adolescents, with only a small minority of about 4% excluded due to reasons such as death, emigration, immigration, or severe intellectual disability. Further, we accessed diagnostic data from both primary and specialist care. Although dichotomous, diagnoses represent mental disorders as defined by professional healthcare workers with specialized training and often formal procedures. The fact that we see the same pattern in diagnoses from both primary and specialist care indicates that the diminishing association with educational performance is not attributable to a greater transfer of healthcare responsibilities from specialist to primary care settings. By utilizing register‐data, we include individuals who are underrepresented in traditional recruitment‐based cohort studies (Brayne & Moffitt, 2022; Fry et al., 2017).

Nevertheless, the results have to be interpreted in light of some limitations. First, due to data constraints, our analyses were restricted to a 13‐year period from 2006 to 2019, which is relatively short in historical terms. Future investigations on a broader timeframe could offer deeper insights into the interplay between mental disorders and academic achievement. Second, the data we have on hand cannot establish a definite causal link between mental disorders and educational performance. Our diagnostic data represent individuals receiving treatment for mental disorders but do not encompass all cases within the population. Lastly, it's important to acknowledge that educational performance and mental health have a bi‐directional relationship and we cannot exclude the possibility that the associations found in this study arise due to poor school performance and not vice versa. Given that the average GPA showed an increase in the time period, we standardized GPA scores within each cohort, and analyses could not be based on absolute GPA scores across the study period.

## CONCLUSION

In conclusion, our study demonstrates a rising prevalence of internalizing disorders and ADHD from 2006 to 2019. We find no evidence to support the idea that the increase in prevalence is predominantly driven by high‐achieving students. Our analyses indicate a gradual decline in the association between these disorders and educational performance. We have discussed several alternative explanations such as the possible benefits of treatment and educational interventions, but place particular emphasis on the hypothesis that the declining associations could reflect shifting diagnostic thresholds. Given an overview of this literature, we caution against uncritically interpreting increasing prevalences as reflecting genuine increases in adolescent mental disorders. In order to ensure the validity and utility of mental health diagnoses, it is crucial for treatment providers to exercise prudence and caution in their diagnostic practices, avoiding excessive use that could potentially undermine the meaningful distinction between normal psychological experiences and clinically significant, pathological conditions.

## AUTHOR CONTRIBUTIONS

**Magnus Nordmo**: Conceptualization; Formal analysis; Investigation; Methodology; Validation; Visualization; Writing – original draft; Writing – review & editing. **Thomas H. Kleppestø**: Conceptualization; Investigation; Methodology; Writing – original draft; Writing – review & editing. **Bjørn‐Atle Reme**: Conceptualization; Investigation; Methodology; Writing – original draft; Writing – review & editing. **Hans Fredrik Sunde**: Conceptualization; Investigation; Methodology; Writing – original draft; Writing – review & editing. **Tilmann von Soest**: Conceptualization; Investigation; Methodology; Writing – original draft; Writing – review & editing. **Fartein Ask Torvik**: Conceptualization; Investigation; Methodology; Supervision; Writing – original draft; Writing – review & editing.

## CONFLICT OF INTEREST STATEMENT

The authors have declared that they have no competing or potential conflicts of interest.

## ETHICAL CONSIDERATIONS

The study was approved by the Regional Committee for Medical and Health Research Ethics.

## Supporting information

Supporting Information S1

## Data Availability

This is a registry study with no data available.

## ENDNOTES

^1^
Given the calculation 0.013 (*interactioncoefficient*)*13(*years*)≈0.17.

^2^
The negative effect of ADHD in 1990 was −1,04 *z*‐scores. To calculate the expected negative effect in 2003 we add the interaction parameter for each of the 13 study years (0.013*13 = 0.17). Next, we add this amount to the 1990 coefficient giving us the predicted difference between 1990 and 2003 (−1.04 + 0.17 = −0.87). Lastly, we calculate the proportion change this implies as $1-\;\frac{-0,87\;\left(2003\right)}{-1,04\;\left(1990\right)}=16\;\%$.

^3^
Same procedure as above with $1-\frac{-0.43\;\left(2003\right)}{-0.59\;\left(1990\right)}=27\;\%$.
