# Supplementary material for: The diminishing association between adolescent mental disorders and educational performance from 2006–2019
Source: JCPP Adv. 2024 May 31;4(3):e12239. doi: 10.1002/jcv2.12239 (PMC11472808; doi:10.1002/jcv2.12239)
Supplement: Supplementary file 1 — Supporting Information S1 [file JCV2-4-e12239-s001.docx]

# Supplementary Appendix:

This appendix provides additional data and methodological details supplementing the primary analyses presented in "The Diminishing Association between Common Adolescent Mental Disorders and Educational Success from 2006 – 2019".

| Supplemental Figure 1: Bivariate Estimates with Specialist Care Diagnoses |
| --- |
| 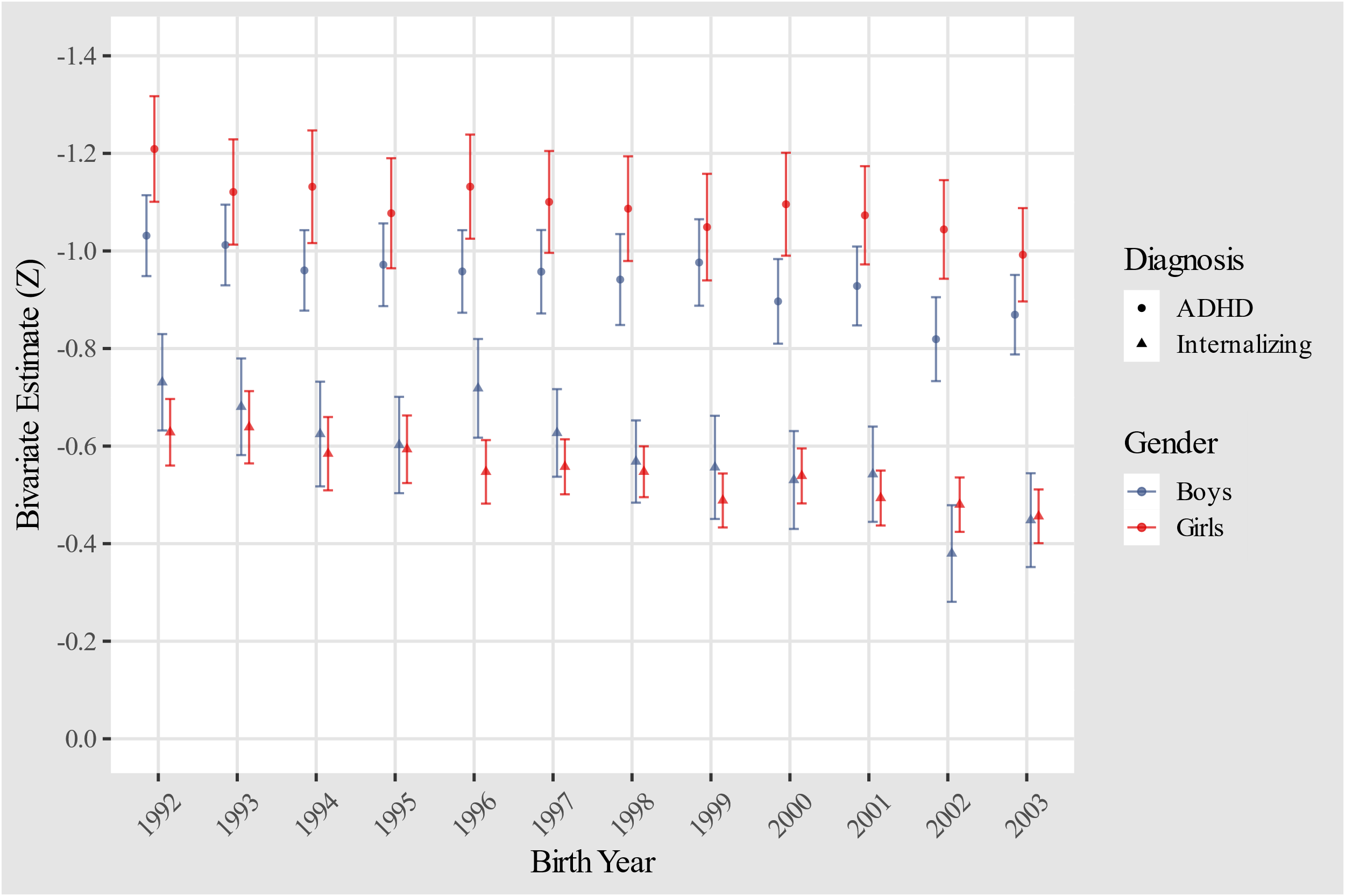 |

In addition to analyses on primary care diagnoses, we also repeated identical models on diagnostic data from specialist care, see Supplemental Figure 1 and Table 1. We found a significant interaction term using these data. When we compare the outcomes from primary and specialist care, we only find a meaningful difference in the main and interaction effect with adolescent internalizing. This is because almost every adolescent with an ADHD diagnosis in primary care, also has a diagnosis in specialist care. This is not the case for internalizing diagnoses where it is common to get a diagnosis from primary care only. The adolescents who only receive a diagnosis from primary care are less severely affected compared with the adolescents who are referred to specialist care.

| Supplemental Table 1: Specialist Care Interaction Models | | | | | |
| --- | --- | --- | --- | --- | --- |
|  | ADHD | |  | Internalizing | |
|  | Coefficient | Standard Error |  | Coefficient | Standard Error |
| \| Intercept \| -0.732 *** \| (0.004) \| \| --- \| --- \| --- \| \| ADHD \| -1.064 *** \| (0.018) \| \| Birth Year \| -0.000 \| (0.000) \| \| Gender \| 0.501 *** \| (0.002) \| \| ADHD × Birth Year \| 0.012 *** \| (0.003) \| \| N \| 752565 \|  \| \| R^2^ \| 0.077 \|  \| | | | \| Intercept \| -0.758 *** \| (0.004) \| \| --- \| --- \| --- \| \| Internalizing \| -0.676 *** \| (0.016) \| \| Birth Year \| 0.000 \| (0.000) \| \| Gender \| 0.518 *** \| (0.002) \| \| Internalizing × Birth Year \| 0.022 *** \| (0.002) \| \| N \| 752565 \|  \| \| R^2^ \| 0.071 \|  \| \|  \| \| \| | | |
| *** p < 0.001; ** p < 0.01; * p < 0.05. | | | | | |

Supplemental Figure 2 shows the yearly average grades for adolescents with and without a diagnosis. The top four panels show GPA measures in raw format, ranging from grade 1 (worst) to grade 6 (best). The upwards slant indicates a rise in average grades over time. This increase is removed by z-standardizing GPA for each year as shown in the bottom four panels.

| Supplemental Figure 2: Average Grades |
| --- |
| 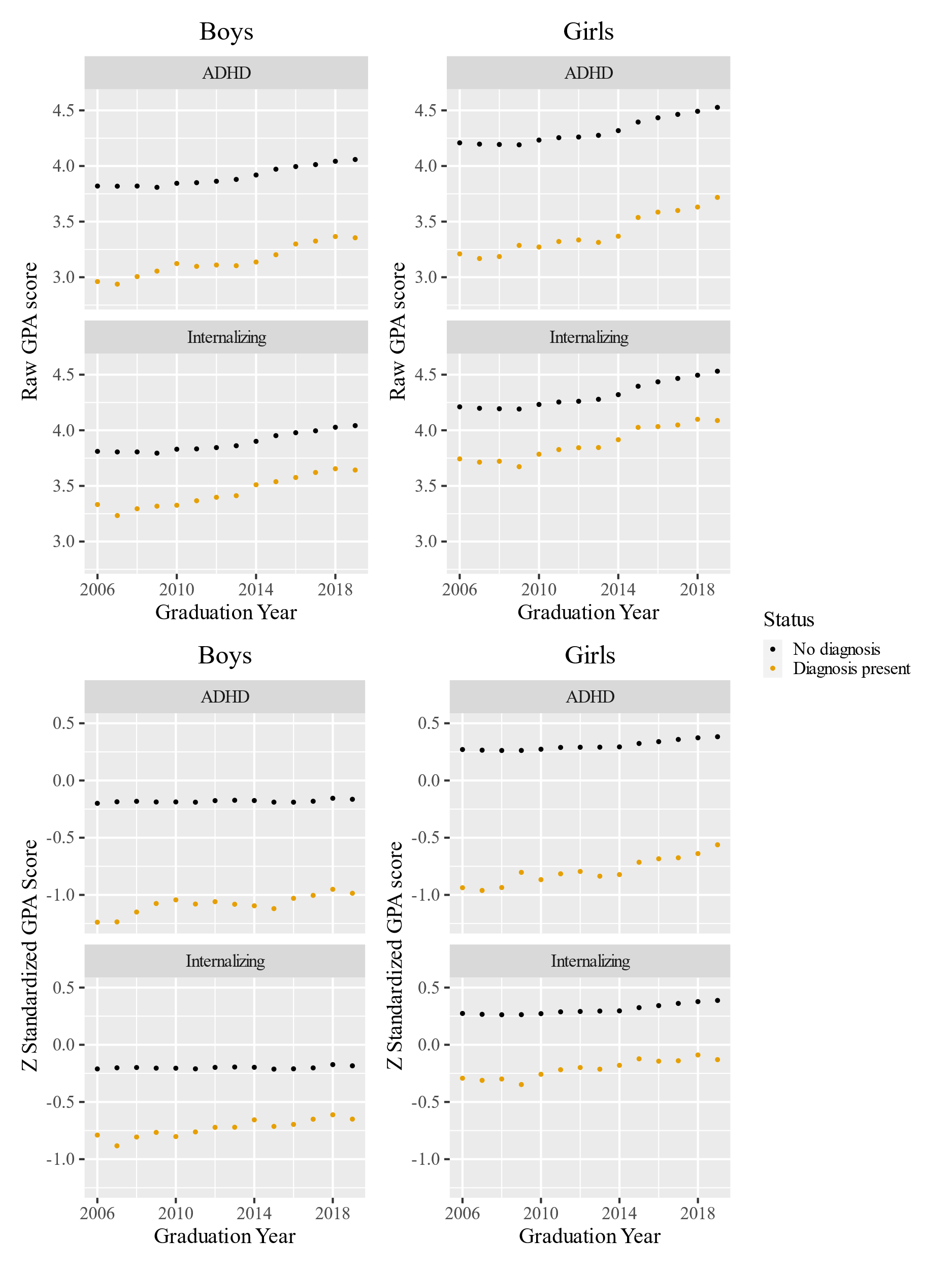 |

See Supplementary Table 2 for primary care prevalences of ADHD and Internalizing in the study period from 2006-2019.

| Supplementary Table 2: Primary Care Diagnostic Prevalence from 2006 – 2019 | | | | | | | | | | |
| --- | --- | --- | --- | --- | --- | --- | --- | --- | --- | --- |
|  | | | Internalizing ^a^ | | | | ADHD ^b^ | | | |
| Graduation Year | N Boys | N Girls | N Disorder Boys | Percent Boys | N Disorder Girls | Percent Girls | N Disorder Boys | Percent Boys | N Disorder Girls | Percent Girls |
| 2006 | 31,009 | 29,634 | 229 | 0.74 | 589 | 1.99 | 462 | 1.49 | 196 | 0.66 |
| 2007 | 31,333 | 29,695 | 224 | 0.71 | 604 | 2.03 | 599 | 1.91 | 257 | 0.87 |
| 2008 | 31,210 | 29,298 | 286 | 0.92 | 632 | 2.16 | 714 | 2.29 | 286 | 0.98 |
| 2009 | 30,575 | 29,352 | 286 | 0.94 | 619 | 2.11 | 744 | 2.43 | 329 | 1.12 |
| 2010 | 30,876 | 29,476 | 297 | 0.96 | 661 | 2.24 | 834 | 2.70 | 334 | 1.13 |
| 2011 | 31,383 | 29,729 | 345 | 1.10 | 775 | 2.61 | 929 | 2.96 | 376 | 1.26 |
| 2012 | 31,836 | 30,155 | 369 | 1.16 | 926 | 3.07 | 995 | 3.13 | 406 | 1.35 |
| 2013 | 31,279 | 29,741 | 417 | 1.33 | 1,059 | 3.56 | 986 | 3.15 | 389 | 1.31 |
| 2014 | 30,427 | 29,245 | 399 | 1.31 | 1,073 | 3.67 | 914 | 3.00 | 384 | 1.31 |
| 2015 | 31,073 | 29,639 | 402 | 1.29 | 1,060 | 3.58 | 999 | 3.22 | 425 | 1.43 |
| 2016 | 31,101 | 29,718 | 444 | 1.43 | 1,172 | 3.94 | 1,006 | 3.23 | 477 | 1.61 |
| 2017 | 29,879 | 28,816 | 444 | 1.49 | 1,167 | 4.05 | 994 | 3.33 | 488 | 1.69 |
| 2018 | 29,471 | 28,459 | 510 | 1.73 | 1,184 | 4.16 | 939 | 3.19 | 428 | 1.50 |
| 2019 | 30,210 | 29,073 | 590 | 1.95 | 1,224 | 4.21 | 1,049 | 3.47 | 521 | 1.79 |
| ^a^ Internalizing = P74, P76, P79 | | | | | | | | | | |
| ^b^ ADHD = P81 | | | | | | | | | | |

See Supplementary Table 3 for specialist care prevalences of ADHD and Internalizing in the study period from 2006-2019.

| Supplementary Table 3: Specialist Care Diagnostic Prevalence from 2006 – 2019 | | | | | | | | | | |
| --- | --- | --- | --- | --- | --- | --- | --- | --- | --- | --- |
|  | | | Internalizing ^a^ | | | | ADHD ^b^ | | | |
| Graduation Year | N Boys | N Girls | N Disorder Boys | Percent Boys | N Disorder Girls | Percent Girls | N Disorder Boys | Percent Boys | N Disorder Girls | Percent Girls |
| 2008 | 32,143 | 30,080 | 384 | 1.19 | 776 | 2.58 | 542 | 1.69 | 302 | 1.00 |
| 2009 | 31,446 | 30,126 | 380 | 1.21 | 660 | 2.19 | 542 | 1.72 | 306 | 1.02 |
| 2010 | 31,854 | 30,279 | 322 | 1.01 | 642 | 2.12 | 543 | 1.70 | 267 | 0.88 |
| 2011 | 32,576 | 30,589 | 377 | 1.16 | 756 | 2.47 | 508 | 1.56 | 280 | 0.92 |
| 2012 | 33,331 | 31,050 | 359 | 1.08 | 860 | 2.77 | 511 | 1.53 | 313 | 1.01 |
| 2013 | 32,749 | 30,727 | 460 | 1.40 | 1,149 | 3.74 | 503 | 1.54 | 325 | 1.06 |
| 2014 | 31,765 | 30,193 | 527 | 1.66 | 1,336 | 4.42 | 427 | 1.34 | 305 | 1.01 |
| 2015 | 32,466 | 30,709 | 332 | 1.02 | 1,164 | 3.79 | 470 | 1.45 | 289 | 0.94 |
| 2016 | 32,637 | 30,883 | 357 | 1.09 | 1,154 | 3.74 | 475 | 1.46 | 320 | 1.04 |
| 2017 | 31,811 | 30,085 | 377 | 1.19 | 1,145 | 3.81 | 547 | 1.72 | 347 | 1.15 |
| 2018 | 32,119 | 29,807 | 372 | 1.16 | 1,143 | 3.83 | 491 | 1.53 | 337 | 1.13 |
| 2019 | 32,607 | 30,533 | 397 | 1.22 | 1,155 | 3.78 | 549 | 1.68 | 371 | 1.22 |
| ^a^ F412,F321,F431,F331,F320,F411,F330,F401,F410,F341,F419 | | | | | | | | | | |
| ^b^ F900,F901 | | | | | | | | | | |
